# Fulminant cerebral edema in the setting of acute dengue fever after mechanical thrombectomy in a patient with massive stroke and severe hypoalbuminemia: a case report

**DOI:** 10.3389/fmed.2026.1759313

**Published:** 2026-05-14

**Authors:** Haiwen Huang, Ying Li, Yonglin Li, Zhihua Liu, Jiajing Hu

**Affiliations:** 1 Boai Hospital of Zhongshan, Zhongshan, Guangdong, China; 2 Zhongshan Traditional Chinese Medicine Hospital, Zhongshan, Guangdong, China; 3 Guangzhou University of Chinese Medicine, Guangzhou, Guangdong, China; 4 Guangdong Second Traditional Chinese Medicine Hospital, Guangzhou, Guangdong, China

**Keywords:** blood–brain barrier, dengue fever, hypoalbuminemia, ischemic stroke, malignant cerebral edema, mechanical thrombectomy

## Abstract

**Background:**

Malignant cerebral edema following mechanical thrombectomy for large vessel occlusion stroke carries high mortality. While reperfusion injury and metabolic factors are established contributors, the potential role of concurrent systemic inflammatory stressors remains underrecognized.

**Case presentation:**

We present the case of a 61-year-old male with a history of nephrotic syndrome and severe hypoalbuminemia (14.8 g/L), who developed an extensive left middle cerebral artery infarction. At approximately 20 h after onset, worsening neurological deficits (NIHSS 9 → 16) prompted mechanical thrombectomy. At 4 h postoperatively, the patient developed an abrupt high fever (39 °C), prompting immediate diagnostic reassessment, which confirmed acute dengue fever (NS1 antigen-positive) and concurrent bacterial pneumonia. Simultaneously, laboratory tests revealed a systemic inflammatory response, with procalcitonin (PCT) peaking at 1.18 ng/mL and markedly elevated interleukin-6 (IL-6). Within the following 20 h, cerebral edema progressed rapidly, with midline shift increasing from 4 mm to 11 mm, consistent with malignant cerebral edema and impending brain herniation. Despite aggressive medical management, the family declined further decompressive surgery and intensive treatment; the patient was discharged against medical advice, and death was confirmed on follow-up.

**Conclusion:**

This report suggests that the fulminant cerebral edema observed in this patient may have been related to blood–brain barrier (BBB) vulnerability following ischemia–reperfusion injury, together with superimposed systemic inflammatory stress and markedly reduced oncotic buffering capacity. Acute dengue infection may have acted as a systemic endothelial and inflammatory stressor during the post-reperfusion vulnerable period, rather than as an independent primary cause. Severe hypoalbuminemia may have represented a baseline physiological susceptibility condition that reduced tolerance to fluid shift once BBB integrity was compromised.

## Introduction

Extensive middle cerebral artery infarction is one of the most severe forms of ischemic stroke. Although endovascular thrombectomy improves recanalization rates, large established infarcts may remain vulnerable to postoperative edema progression. Wang et al. (1) and Pu et al. (2) have shown that malignant cerebral edema after mechanical thrombectomy or successful reperfusion is itself an established and clinically important complication, supporting the concept of a vulnerable post-reperfusion window. This case suggests that during the post-reperfusion period of blood–brain barrier vulnerability, cerebral edema progression may be influenced by multiple interacting systemic factors. In parallel, Trivedi and Chakravarty (3) noted that dengue-associated neurological deterioration may occur in the setting of systemic inflammation, capillary leakage, and cerebral edema, rather than only through direct neuroinvasion, while Guzman and Martinez (4) further emphasized that the neurological spectrum of dengue extends beyond encephalitis to broader systemic and cerebrovascular manifestations. In particular, acute dengue infection may act as a systemic endothelial and inflammatory stressor, contributing to increased blood–brain barrier permeability in a context already characterized by ischemia–reperfusion injury and severe hypoalbuminemia. Severe hypoalbuminemia may further reduce physiological tolerance to fluid shift by lowering plasma oncotic buffering capacity. These observations suggest that the coexistence of these factors may have been related to the unusually rapid and non-linear progression of cerebral edema in this patient. Thus, the present case is distinctive in that these otherwise separately described processes appeared to converge within the same postoperative time window, resulting in an unusually rapid and non-linear progression of cerebral edema. Herein, we report a rare case of concurrent dengue infection, hypoalbuminemia, and malignant post-thrombectomy cerebral edema, culminating in rapid neurological deterioration within 48 h. In post-thrombectomy patients with extensive infarction, clinicians should maintain heightened vigilance when systemic inflammatory features such as fever occur, particularly in dengue-endemic regions, where early recognition of concurrent infection may facilitate timely diagnostic evaluation and supportive management.

## Case report

**Figure 1:**
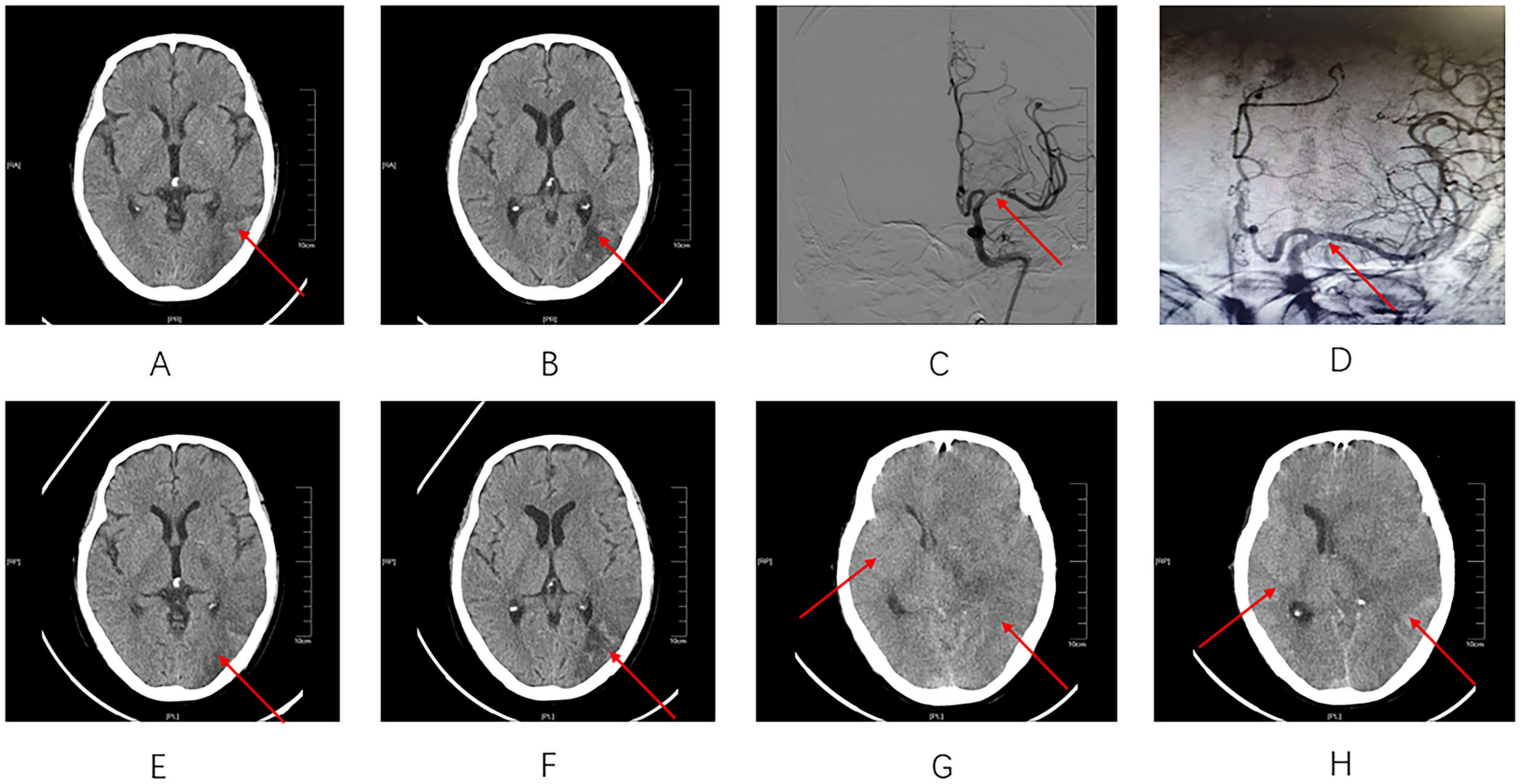
Serial neuroimaging before and after mechanical thrombectomy. **(A)** Axial CT shows a large hypodense area in the left fronto-temporo-parietal region (arrow), consistent with an extensive infarction in the left MCA territory. No obvious midline shift is observed at this stage. **(B)** A slightly higher axial level again demonstrates low attenuation in the left cerebral hemisphere with cortical sulcal effacement (arrow), but no obvious midline shift at this stage. **(C)** Left internal carotid angiogram shows distal MCA occlusion/severe stenosis with poor distal filling (arrow). **(D)** Post-thrombectomy angiogram demonstrates restored antegrade flow in the left MCA with re-opacification of distal branches (arrow). **(E)** Axial CT shows increased edema of the infarcted left cerebral hemisphere with sulcal effacement. A focal hyperdense area is seen within the infarct zone (arrow), suggesting contrast extravasation or mild hemorrhagic transformation in the early post-reperfusion stage. **(F)** At a slightly different level, the same linear/wedge-shaped hyperdensity is again demonstrated (arrow), accompanied by compression of the left lateral ventricle and a mild rightward midline shift, indicating a vulnerable blood–brain barrier after endovascular therapy. **(G)** Diffuse swelling of the left cerebral hemisphere with effacement of the cortical sulci, marked compression of the left lateral ventricle, and rightward midline shift. **(H)** Further progression of hemispheric edema with near-complete obliteration of the left lateral ventricle and a marked nonlinear increase in rightward midline shift to 11 mm within 20 h of the previous scan, consistent with malignant cerebral edema and impending brain herniation.

A 61-year-old male patient was admitted to the emergency department with right-sided limb weakness for 1 day, followed by worsening confusion and slurred speech for 2 h. He had a history of membranous nephropathy with persistent 3+ proteinuria, for which he had not received regular systematic treatment, and had intermittent lower limb edema. Epidemiological history revealed that one family member had been diagnosed with dengue fever within the preceding week. Before stroke presentation, there was no documented fever or other clear viral prodromal symptoms, and the patient’s initial presentation was dominated by acute focal neurological deficits. On admission examination, the patient appeared somnolent, with equal and reactive pupils, preserved extraocular movements, symmetrical forehead creases and nasolabial folds, tongue in the midline, left-sided limb strength grade 4, right-sided limb strength grade 3, intact superficial and deep sensation, a positive right Babinski sign, and no meningeal irritation signs. The NIHSS score was 9, with mild pitting edema of the face, abdomen, and both lower limbs. Emergency cranial CT revealed extensive low-density areas in the left middle cerebral artery territory, with midline structures largely preserved. The CT revealed scattered punctate hyperdense foci within the infarct, consistent with early hemorrhagic transformation (Figures 1A,B). Laboratory investigations revealed an extremely high-risk metabolic profile: markedly low serum albumin (14.8 g/L) and elevated low-density lipoprotein cholesterol (LDL-C) at 5.43 mmol/L. Albumin supplementation and neuroprotective therapy were initiated immediately upon admission.

On the second day of hospitalization (approximately 20 h post-onset), the patient’s condition deteriorated abruptly. Neurological function rapidly worsened: consciousness further declined, right-sided upper and lower limb strength decreased to grade 0, and the NIHSS score progressed from 9 to 16. Emergency DSA revealed severe middle cerebral artery stenosis (Figure 1C). Intraoperatively, the SWIM technique was employed to advance the intermediate catheter proximally to the thrombus. Combined stent-retriever and aspiration technique (Solumbra technique) was then performed, restoring blood flow postoperatively (TICI 2b/3) (Figure 1D). Intraoperatively, the patient developed sudden dyspnea and tachycardia, which were considered suggestive of a contrast-induced allergic reaction (CEAR), for which dexamethasone and aminophylline were administered symptomatically.

Four hours postoperatively, the patient’s temperature abruptly rose to 39 °C. Repeated laboratory tests revealed a systemic inflammatory response, with procalcitonin (PCT) elevated to 1.18 ng/mL and markedly increased interleukin-6 (IL-6). Urgent dengue antigen testing returned NS1 positive, confirming acute dengue fever. Concurrent sputum culture revealed *Staphylococcus aureus* (4+), confirming concurrent bacterial pneumonia. At this time, the patient remained somnolent, with occasional spontaneous lifting of the right limbs and equal, reactive pupils. A repeat cranial CT (Figures 1E,F) demonstrated worsening edema within the infarct zone with band-like hyperdense areas, intracranial pneumocephalus, and a mild midline shift to the right by approximately 4 mm. Follow-up chest CT also demonstrated a dynamic increase in bilateral pleural effusion, supporting the presence of a systemic leakage process. Given the coexistence of systemic edema and high fever, key differential diagnoses were immediately evaluated: First, cardiac causes of systemic edema were considered less likely because bedside echocardiography revealed a left ventricular ejection fraction (LVEF) of 65%, arguing against hydrostatic edema due to cardiac pump failure. Second, dengue hemorrhagic fever (DHF) was considered less likely because the patient’s platelet count remained within the normal range throughout (190–251 × 10^9^/L), arguing against diffuse bleeding due to coagulation disorders.

Immediate treatment commenced with cefoperazone-sulbactam for infection control, continuous albumin supplementation, and anti-edema therapy. Cooling measures were initiated, dexamethasone was continued, dehydration therapy with mannitol and albumin was intensified, blood pressure was controlled, and supportive treatment was provided. Nevertheless, the patient’s neurological status continued to deteriorate rapidly. Between 27 and 44 h after onset, the patient developed progressive neurological deterioration, with consciousness worsening to superficial coma and both pupils measuring 4 mm with sluggish light reflexes. On postoperative day 2 (44 h post-onset), repeat cranial CT (Figures 1G,H) revealed diffuse cerebral edema, complete occlusion of the left lateral ventricle, and a midline shift progressing from 4 mm to 11 mm within approximately 20 h.

The patient was subsequently transferred to the intensive care unit for continued dehydration, cooling, and respiratory support. Decompressive craniectomy was recommended; however, the family declined further surgical decompression and intensive treatment, and the patient was discharged against medical advice later that same day. Post-discharge follow-up subsequently confirmed death. For clarity, the major clinical events, neurological findings, imaging/laboratory changes, and therapeutic interventions in this case are summarized chronologically in Table 1.

**Table 1:** Chronological clinical summary of the case.

Time point	Clinical/neurological findings	Key investigations	Main interventions/clinical decisions
At admission	Somnolent; pupils equal and reactive; extraocular movements preserved; symmetrical forehead creases and nasolabial folds; tongue midline; left limb strength grade 4, right limb strength grade 3; intact superficial and deep sensation; right Babinski sign positive; no meningeal irritation signs; NIHSS 9; mild pitting edema of face, abdomen, and both lower limbs	Cranial CT: extensive low-density lesion in the left MCA territory with largely preserved midline structures; scattered punctate hyperdense foci consistent with early hemorrhagic transformation (Figures 1A,B). Laboratory tests: albumin 14.8 g/L; LDL-C 5.43 mmol/L	Albumin supplementation and neuroprotective therapy initiated
Approximately 20 h post-onset	Abrupt neurological deterioration; consciousness further declined; right upper and lower limb strength decreased to grade 0; NIHSS increased from 9 to 16	Emergency DSA: severe middle cerebral artery stenosis (Figure 1C)	Emergency mechanical thrombectomy performed
Intraoperatively	Sudden dyspnea and tachycardia, suggestive of contrast-induced allergic reaction	Angiographic recanalization achieved after thrombectomy (TICI 2b/3) (Figure 1D)	SWIM + Solumbra technique; symptomatic treatment with dexamethasone and aminophylline
4 h postoperatively	High fever (39 °C); remained somnolent; occasional spontaneous lifting of the right limbs; pupils equal and reactive	PCT 1.18 ng/mL; IL-6 markedly elevated; dengue NS1 antigen positive; sputum culture: *Staphylococcus aureus* (4+). Cranial CT: worsening edema, band-like hyperdense areas, intracranial pneumocephalus, and mild midline shift (~4 mm) (Figures 1E,F). Follow-up chest CT: progressive bilateral pleural effusion.	Cooling measures; dexamethasone; intensified dehydration therapy with mannitol and albumin; blood pressure control; cefoperazone-sulbactam; supportive treatment
27–44 h post-onset	Progressive neurological deterioration; consciousness worsened to superficial coma; both pupils 4 mm with sluggish light reflexes	Clinical progression	Continued anti-edema, anti-infective, cooling, and supportive treatment
44 h post-onset (postoperative day 2)	Severe neurological deterioration	Cranial CT: diffuse cerebral edema, complete occlusion of the left lateral ventricle, and midline shift progressing from 4 mm to 11 mm within approximately 20 h (Figures 1G,H)	Transferred to ICU for continued dehydration, cooling, and respiratory support; decompressive craniectomy recommended
Final outcome	Progressive malignant cerebral edema	—	Family declined further surgical decompression and intensive treatment; patient discharged against medical advice the same day; death subsequently confirmed on follow-up.

## Discussion

Extensive middle cerebral artery (MCA) infarction represents one of the most severe forms of ischemic stroke (5, 6). Previous studies indicate that the period between 24 and 72 h post-onset constitutes a critical window for progressive cerebral edema and midline shift. Once malignant cerebral edema develops, it frequently progresses rapidly to brain herniation, carrying a poor prognosis. Recently, endovascular thrombectomy has significantly improved recanalization rates for large vessel occlusions (7, 8). However, for established extensive infarcts, reperfused brain tissue remains in a ‘susceptible phase’ prone to edema (9). If this phase is compounded by factors increasing capillary permeability—such as acute dengue fever, postoperative pulmonary infection, or hypoalbuminemia—the onset and progression of cerebral edema may be further amplified, potentially manifesting as progressive intracranial hypertension occurring faster than within the typical time window. However, reperfusion injury alone may not fully explain such rapid worsening of cerebral edema within 20 h, with the midline shift increasing from 4 mm to 11 mm. This suggests that additional systemic modifying factors may have contributed to the clinical course.

Dengue fever is a common mosquito-borne viral infection in southern China and other tropical and subtropical regions (10). Recent studies indicate that dengue fever may involve the central or peripheral nervous system in some patients, with neurological involvement often signifying more severe disease (11). Dengue-associated neurological manifestations are commonly classified into three categories: encephalitic manifestations related to direct viral invasion, encephalopathic manifestations related to systemic factors such as high fever and increased vascular permeability, and post-infectious immune-mediated manifestations (12). Among these, ‘dengue encephalopathy’ is the most prevalent. Its core mechanism is believed to involve cerebral edema resulting from systemic inflammatory responses and capillary leakage, potentially amplifying pre-existing cranial brain injury (13).

We reviewed recent literature on dengue-associated neurological involvement and malignant edema of the brain following mechanical thrombectomy, finding no reports entirely consistent with this case (14, 15). This patient presented with a vulnerable blood–brain barrier following thrombectomy, compounded by a systemic inflammatory response triggered by acute NS1-positive dengue fever, concurrent bacterial pulmonary infection, and profound hypoalbuminemia associated with nephrotic syndrome. This combination of post-thrombectomy BBB vulnerability, systemic inflammatory activation, and profound hypoalbuminemia was temporally associated with a rapid worsening of cerebral edema, with midline shift increasing from 4 mm to 11 mm within approximately 20 h.

The period between 24 and 72 h after onset represents the peak window for progressive cerebral edema in extensive middle cerebral artery infarction (16, 17). In this case, a midline shift of approximately 4 mm, minor hemorrhagic transformation, and contrast extravasation were observed on the afternoon postoperative CT scan, indicating that the brain tissue had already entered this characteristic vulnerable period. Although the patient experienced a suspected allergic reaction intraoperatively, and postoperative CT revealed band-like hyperdense areas suggestive of contrast-induced encephalopathy (CIE) as a possible early contributor to diffuse edema, the subsequent clinical course did not conform to typical CIE. CIE is generally transient and reversible with symptomatic management, whereas this patient developed irreversible and progressive intracranial decompensation. Furthermore, follow-up chest CT showed a dynamic increase in bilateral pleural effusion, supporting a systemic leakage process beyond isolated CIE. Differential evaluation further supported this interpretation: bedside echocardiography revealed an LVEF of 65%, arguing against hydrostatic edema due to heart failure, while the patient’s consistently normal platelet count (190 × 10^9^/L) argued against diffuse hemorrhage related to dengue hemorrhagic fever. Notably, the severe hypoalbuminemia (14.8 g/L) in this case should be understood in the context of chronic membranous nephropathy with persistent proteinuria, rather than as an isolated acute abnormality or a purely incidental laboratory finding. This nephrotic background likely reflected markedly reduced plasma oncotic buffering capacity before postoperative deterioration occurred. The resulting pre-existing state of critical colloid osmotic pressure (COP) deficiency represented a physiological susceptibility condition, reducing intracranial tolerance to fluid extravasation once the blood–brain barrier was compromised. In this setting, the chronic nephrotic background was clinically relevant because it may have reduced tolerance to fluid shift and facilitated edema progression once blood–brain barrier integrity was compromised.

Primary dengue meningoencephalitis was also considered in the differential diagnosis; however, it was regarded as less likely to be the principal explanation in this case. The patient initially presented with an acute focal stroke syndrome rather than a meningoencephalitic syndrome, and there was no documented fever or other clear viral prodromal symptoms before presentation. In addition, no meningeal irritation signs were present on admission. High fever and NS1 positivity were identified only postoperatively, which further supports the interpretation that acute dengue infection acted as a superimposed systemic inflammatory/endothelial stressor during the post-reperfusion vulnerable period, rather than as a proven initiating neurological cause. No cerebrospinal fluid examination was performed because lumbar puncture was considered clinically contraindicated in the setting of rapidly progressive cerebral edema, marked mass effect, and a high risk of brain herniation.

**Figure 2:**
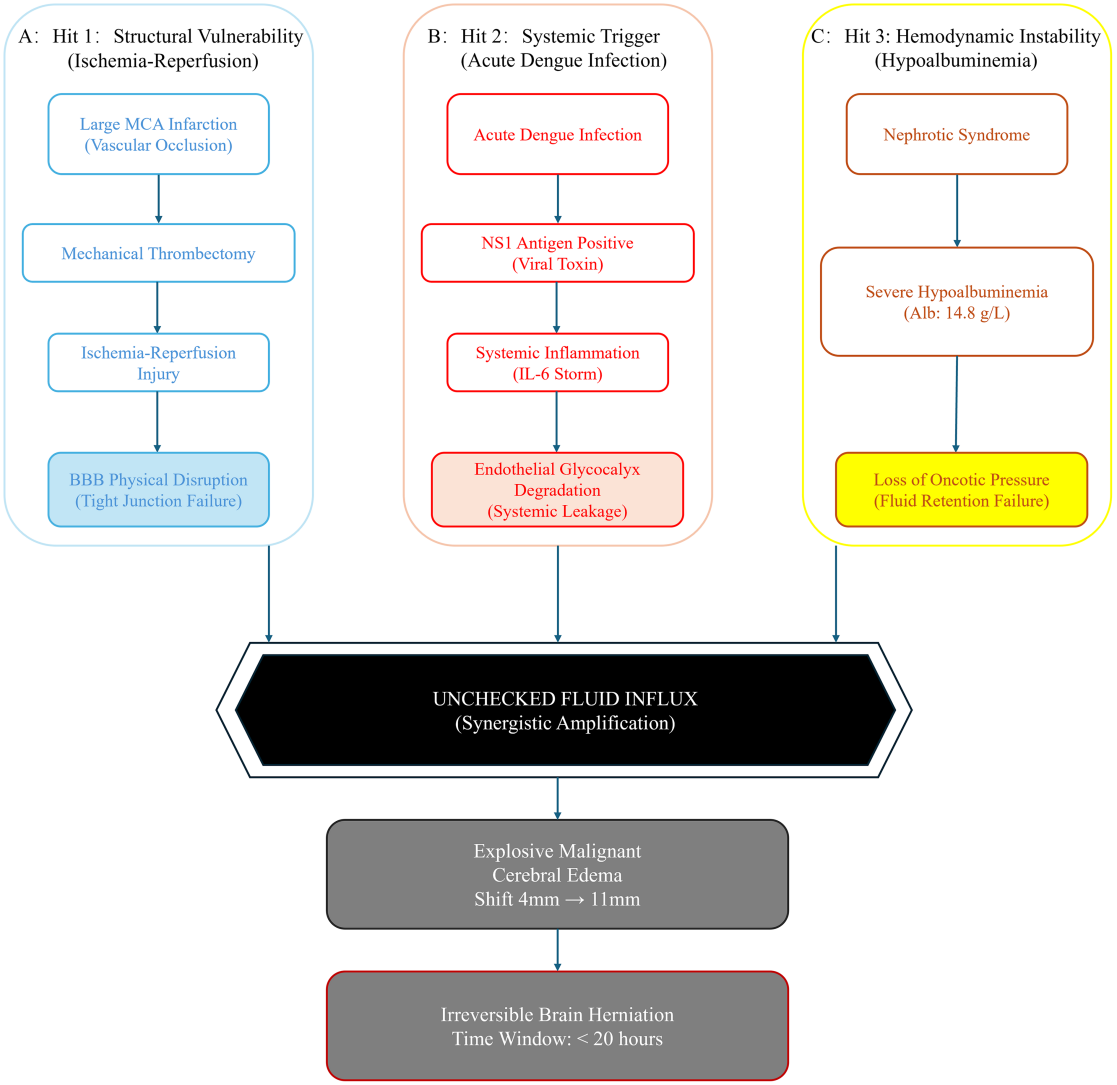
Schematic illustration of a proposed conceptual model derived from clinical reasoning to interpret the fulminant cerebral edema observed in this case. The schematic depicts three contributing components: **(A)** Structural vulnerability, in which large-vessel infarction and subsequent thrombectomy reperfusion may compromise blood–brain barrier (BBB) integrity; **(B)** systemic inflammatory stress, in which acute dengue infection, evidenced by NS1 antigen positivity, may be associated with endothelial activation and increased vascular permeability; and **(C)** physiological susceptibility, in which severe hypoalbuminemia (14.8 g/L) is associated with reduced plasma oncotic buffering capacity. This schematic is intended as a hypothesis-generating clinical interpretation and does not represent a validated mechanistic pathway.

The clinical course in this case was characterized by irreversible and progressive intracranial decompensation, temporally accompanied by postoperative fever (39 °C) and elevation of PCT (1.18 ng/mL). In this report, the postoperative inflammatory surge together with the positive NS1 antigen result was considered more consistent with acute dengue infection acting as a superimposed systemic endothelial and inflammatory stressor during the post-reperfusion vulnerable period, rather than as an independent primary cause. Accordingly, we present Figure 2 as a conceptual interpretation derived from clinical reasoning based on the clinical findings in this case, rather than as a validated mechanistic pathway. Among dengue-associated neurological sequelae, the temporal sequence observed in this patient appears to be more consistent with previously reported patterns of cerebral dysfunction associated with systemic dengue infection (18–20). The underlying pathophysiological substrate is characterized by ischemia–reperfusion–related structural disruption of the blood–brain barrier combined with critically reduced plasma oncotic buffering capacity due to severe hypoalbuminemia (14.8 g/L). Within this pre-existing vulnerability state, systemic inflammatory stress associated with dengue infection may have contributed to further blood–brain barrier instability and to the unusually rapid, non-linear progression of cerebral edema, which typically develops over 24–72 h but in this case evolved within approximately 20 h. This marked escalation from 4 mm to 11 mm occurred in the context of preserved cardiac function (EF 65%) and was temporally associated with systemic inflammatory activation. Importantly, NS1 antigen positivity is interpreted here as evidence of systemic endothelial activation rather than a primary neurological driver. The interaction between blood–brain barrier structural vulnerability and severe hypoalbuminemia-associated loss of oncotic buffering capacity likely created a permissive physiological environment for fluid shift into the cerebral compartment. This may also have contributed to the limited response to standard hyperosmolar therapy and to the unusually rapid progression of cerebral edema in this case. Accordingly, hypoalbuminemia was not interpreted as a direct causal trigger of the acute event, but rather as a chronic background condition that may have reduced tolerance to fluid shift in the setting of post-reperfusion BBB vulnerability and systemic inflammatory stress. In this context, dengue infection should be understood as a modifying systemic stressor that may have further promoted decompensation, rather than a causal initiating factor. Finally, this refractory clinical course highlights the severity of combined systemic and neurological stressors in this setting, despite aggressive intervention.

Differences from Previous Reports and Clinical Implications Previously reported cases of dengue-associated central nervous system involvement predominantly occurred in patients without pre-existing significant cranial organic lesions, with marked midline shift or even brain herniation rarely developing within 24 h (21). Similarly, studies in the stroke field seldom include concurrent viral fever or hypoalbuminemia among precipitating factors. The peculiarity of this case lies in the temporal overlap between the extensive infarct edema window and the systemic leak phase associated with acute dengue infection. We propose that the synchronous convergence of these factors may help explain the unusually rapid increase in midline shift from 4 mm to 11 mm within approximately 20 h.

In summary, this case suggests several precautionary considerations for similar high-risk clinical scenarios. In dengue-endemic regions, early dengue screening may be considered in post-thrombectomy patients who develop atypical fever within 24 h. For patients with extensive infarction and systemic inflammatory features, shorter intervals between cranial CT examinations during the 24–72-h high-risk edema window may be prudent. In addition, correction of profound hypoalbuminemia may help improve physiological tolerance to fluid shifts, although this remains a supportive rather than disease-specific intervention. Finally, this fatal course underscores the importance of early communication with patients and families regarding the risk of fulminant deterioration and the timing of potential decompressive surgery. These considerations are derived from a single case and should not be interpreted as established standards of care.

## Data Availability

The raw data supporting the conclusions of this article will be made available by the authors, without undue reservation.
